# High survival rate of 43% in out-of-hospital cardiac arrest patients in an optimised chain of survival

**DOI:** 10.1007/s12471-014-0617-x

**Published:** 2014-10-18

**Authors:** L. W. Boyce, T. P. M. Vliet Vlieland, J. Bosch, R. Wolterbeek, G. Volker, H. J. van Exel, C. Heringhaus, M. J. Schalij, P. H. Goossens

**Affiliations:** 1 Rijnlands Rehabilitation Centre, Wassenaarseweg 501, 2333AL Leiden, the Netherlands; 2 Department of Orthopaedics, Leiden University Medical Center, Albinusdreef 2, 2333 ZA Leiden, the Netherlands; 3 Emergency Medical Service Hollands Midden, Vondellaan 43, 2332 AA Leiden, the Netherlands; 4 Department of Medical Statistics, Leiden University Medical Center, Einthovenweg 20, 2333 ZC Leiden, the Netherlands; 5 Department of Cardiology, Leiden University Medical Center, Albinusdreef 2, 2333 ZA Leiden, the Netherlands; 6 Department of Accident and Emergency, Leiden University Medical Center, Albinusdreef 2, 2333 ZA Leiden, the Netherlands; 7 Department of Rehabilitation, Leiden University Medical Center, Albinusdreef 2, 2333 ZA Leiden, the Netherlands; 8 Rijnlands Rehabilitation Centre, PO Box 176, 2300 AD Leiden, the Netherlands

**Keywords:** Out of hospital cardiac arrest, Survival, Cardiopulmonary resuscitation

## Abstract

**Aims:**

Survival to hospital discharge after out-of-hospital cardiac arrest (OHCA) varies widely. This study describes short-term survival after OHCA in a region with an extensive care path and a follow-up of 1 year.

**Methods:**

Consecutive patients ≥16 years admitted to the emergency department between April 2011 and December 2012 were included. In July 2014 a follow-up took place. Socio-demographic data, characteristics of the OHCA and interventions were described and associations with survival were determined.

**Results:**

Two hundred forty-two patients were included (73 % male, median age 65 years). In 76 % the cardiac arrest was of cardiac origin and 52 % had a shockable rhythm. In 74 % the cardiac arrest was witnessed, 76 % received bystander cardiopulmonary resuscitation and in 39 % an automatic external defibrillator (AED) was used. Of the 168 hospitalised patients, 144 underwent therapeutic procedures. A total of 105 patients survived until hospital discharge. Younger age, cardiac arrest in public area, witnessed cardiac arrest, cardiac origin with a shockable rhythm, the use of an AED, shorter time until return of spontaneous circulation, Glasgow Coma Scale (GCS) ≥13 during transport and longer length of hospital stay were associated with survival. Of the 105 survivors 72 survived for at least 1 year after cardiac arrest and 6 patients died.

**Conclusion:**

A survival rate of 43 % after OHCA is achievable. Witnessed cardiac arrest, cardiac cause of arrest, initial cardiac rhythm and GCS ≥13 were associated with higher survival.

## Introduction

Out-of-hospital cardiac arrest (OHCA) is one of the main causes of death in Europe. A systematic review including 67 peer-reviewed studies published from 1990 to 2008 concludes that the incidence of emergency medical service (EMS) attended OHCA in Europe is 86.4 per 100,000 inhabitants per year [1]. That review reports that 60 % of the patients in Europe are treated by EMS after OHCA and 9 % of these patients survive to hospital discharge. In the Netherlands survival rates seem to be relatively high: a study on EMS-attended OHCA between 2005 and 2008 reported a survival rate until discharge of 14 % [2]. In 1998, a survival rate of 36 % for EMS-attended OHCA was found in the Amsterdam region [3]. However, that study only included patients who had had an OHCA due to a primary cardiac cause.

Factors positively associated with short-term survival after OHCA described in two systematic reviews are: younger age, male gender, witnessed OHCA, early start of cardiopulmonary resuscitation (CPR), initial rhythm of ventricular fibrillation (VF), the use of an automatic external defibrillator (AED), short time until arrival of ambulance, no EMS intubation and short time until return of spontaneous circulation (ROSC) [4, 5]. In-hospital factors important for survival include therapeutic hypothermia and the availability for acute cardiac interventions 24/7 [6, 7]. Revascularisation procedures and the use of an implantable cardioverter defibrillator (ICD) mainly reduce long-term mortality [8].

In the Leiden region efforts are taken to provide an optimal chain for OHCA patients, including optimisation of acute care, treatment during transport, treatment in hospital and cardiac rehabilitation. This study describes survival in an optimised chain for OHCA patients and a follow-up of at least 1 year.

## Methods

In the Leiden area post-cardiac arrest care is organised around one regional cardiac centre (The Leiden University Medical Center, LUMC), where cardiac procedures can be performed 24/7. This centre has an affiliated area of 540 km^2^ with 542,000 inhabitants. [Statistics Netherlands 2012, www.cbs.nl]. The 112 emergency service alerts the regional ambulance service and other first responders, all equipped with an AED and trained personnel.

Chest compressions performed by the ambulance service are standardised using the Lund University Cardiac Arrest System (LUCAS™, Jolife AB/Physio-Control Lund, Sweden). The ambulance service transports all patients with a chance of survival to the emergency department (ED) of the LUMC (approximately 70 % of the cases; personal communication). If ROSC is achieved, patients are transported to the coronary care unit (CCU) or intensive care unit (ICU). In accordance with guidelines, eligible patients receive mild hypothermia.

This retrospective study included patients ≥16 years, resuscitated outside the hospital and admitted to the ED of the LUMC (April 2011–January 2013). In July 2014 a follow-up took place, including only patients who were discharged alive and who had follow-up treatment in the LUMC. Eligible patients were identified using the electronic diagnosis registry of the LUMC. To ensure no patients were missed, patient selection was checked with the registries of the ambulance service. Patients were excluded if the ambulance service decided not to transport the patient to the ED, if the collapse was not caused by cardiac arrest or when insufficient information (<50 %) was available from the medical records.

Data were retrieved using a standardised form. A second researcher checked the data of 10 % randomly selected patients. No major differences were found.

The following data were extracted from the medical records: socio-demographic data (gender and age at time of the cardiac arrest); characteristics of the cardiac arrest: cardiac or non-cardiac (trauma, drowning, intoxication, asphyxia, hypovolaemia or other), home or public area (street, work, sports, ambulance), witnessed or not, CPR bystander or first responder and use of AED; treatment and course (the number of shocks provided by EMS), initial cardiac rhythm (shockable or non-shockable) and the interval between collapse and ROSC; the Glasgow Coma Scale (GCS) during ambulance transport or if not available at arrival to the ED; (sub)acute treatment in the hospital, number of days in hospital, hospital survival and discharge destination. Data for the follow-up were extracted from the medical records (LUMC): Survival (dead or alive); if alive: time since cardiac arrest (months); if deceased: time from cardiac arrest until death (days).

All statistical analyses were performed using the SPSS 19 software package. Descriptive statistics were used for the characteristics of the participants. Characteristics of survivors and non-survivors were compared by unpaired t-tests (Mann–Whitney U test) or Chi square tests, where appropriate. Factors associated with survival (*p* < 0.05) were entered into bivariate and multivariate logistic regression analyses, with survival until hospital discharge as dependent variable. By backward elimination, variables that lacked independent association were removed.

This retrospective study (chart review only) falls outside the remit of the Dutch Medical Research Involving Human Beings Act (Medical Ethical Review Board of the LUMC).

## Results

### Patients

**Fig. 1:**
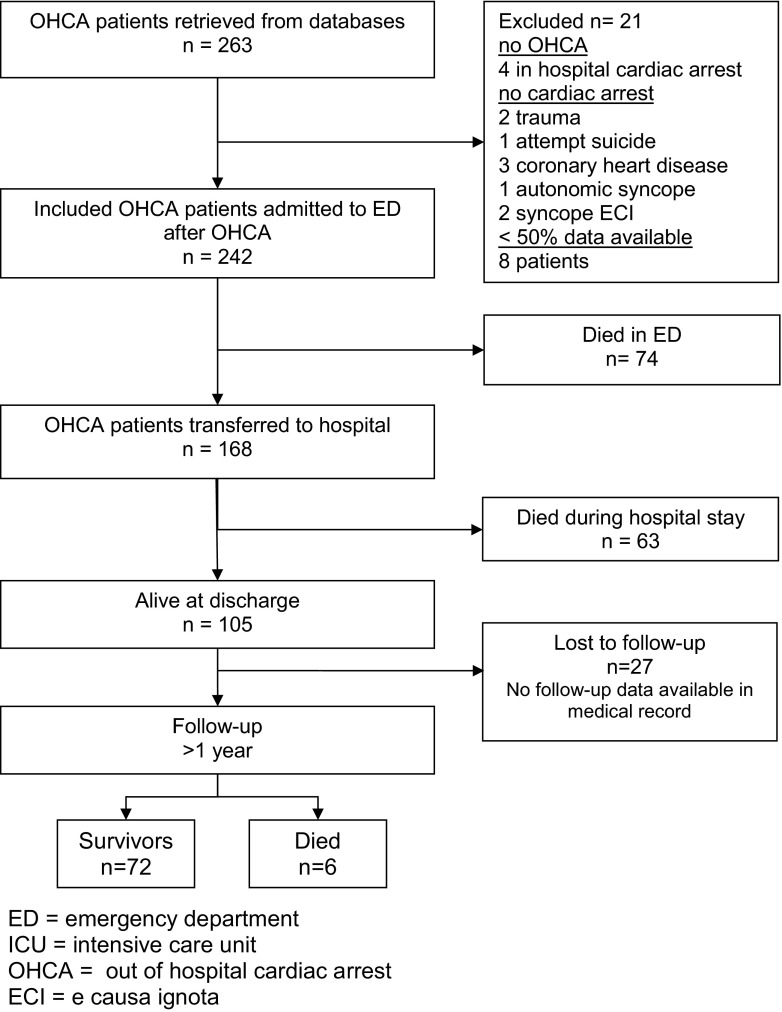
Flow chart of patients after OHCA

In the study period 263 patients were identified. After examination of the medical records 21 patients were excluded (Fig. 1).

Table 1 shows the socio-demographic characteristics, characteristics of the medical condition and treatment and course of the 242 included patients. Their median age was 65 years (range 20–95) and 73 % were male. Of the OHCAs 67 % were witnessed by bystanders and 9 % by EMS personnel. A total of 76 % of the patients received bystander CPR and in nearly 40 % an AED was used. Most of the arrests took place at home. The majority of the cardiac arrests were of cardiac origin with a shockable rhythm in about half of all patients at EMS arrival.

**Table 1:** Characteristics of 242 patients who survived out-of-hospital cardiac arrest

	All patients *n* = 242		Survivors *n* = 105 (43 %)		Non-survivors *n* = 137 (57 %)		*P* value^a^
	n		n		n		
Age in years, mean (SD)		64.8 (14.7)		61.5 (13.7)		67.4 (15)	.002
Male gender, n (%)		176 (73)		77 (73)		99 (72)	.853
Location of CA, n (%)							
In/around home	241	148 (61)	105	47 (45)	136	101 (74)	<.001
Public area		93 (39)		58 (55)		35 (26)	<.001
Cause CA, n (%)							
Cardiac	240	183 (76)	104	93 (89)	136	90 (66)	<.001
Non-cardiac		57 (24)		11 (11)		46 (34)	<.001
Witnessed CA, n (%)							
Yes	237	159 (67)	103	82 (80)	134	77 (57)	<.001
CPR, n (%)							
CPR bystander	240	123 (51)	104	57 (55)	136	66 (49)	.335
CPR first responder		61 (25)		27 (26)		34 (25)	.865
Emergency medical service		56 (23)		20 (19)		36 (27)	.189
AED, n (%)							
Yes	240	94 (39)	104	53 (51)	136	41 (30)	.001
Monitored arrest, n (%)							
Yes	242	21 ( 9)	105	11 (11)	137	10 (7)	.384
Initial cardiac rhythm, n (%)							
Shockable	237	145 (61)	102	88 (86)	135	57 (42)	<.001
Non-shockable		92 (39)		14 (14)		78 (58)	<.001
Number of defibrillations		2.5 (±3)		2.3 (±2)		2.7 (±3.5)	.326
Interval collapse – ROSC, n (%)							
No ROSC	221	35 (16)	95	0 (0)	126	35 (28)	<.001
<6 min.		74 (33)		62 (65)		12 (10)	<.001
6–10 min.		18 ( 8)		10 (11)		8 (6)	.261
>10 min.		94 (43)		23 (24)		71 (56)	<.001
Glasgow Coma Scale, n (%)							
Minor ≥13	196	14 (7)	69	13 (19)	127	1 (1)	<.001
Moderate 9–12		6 (3)		5 (7)		1 (1)	.012
Severe <9		176 (90)		51 (74)		125 (98)	<.001
Cardiac intervention *, n (%)							
PCI	242	73 (30)	105	55 (52)	137	18 (13)	
CABG		12 ( 5)		11 (10)		1 (1)	
ICD		26 (11)		26 (25)		0 (0)	
Therapeutic hypothermia		94 (39)		49 (47)		45 (33)	
Length hospital stay (days)		5 (±8.2)		9 (±8.7)		2 (±6.3)	<.001

^a^ P-value with Chi-square or T-test

* Patients could undergo more than one (sub)acute intervention

*CA* Cardiac arrest, *CPR* Cardiopulmonary resuscitation, *AED* Automatic external defibrillator, *ROSC* Return of spontaneous circulation, *PCI* Percutaneous coronary intervention, *CABG* Coronary artery bypass graft, *ICD* Implantable cardioverter defibrillator

### Survival and discharge destination

Of the 242 patients who attended the ED, 74 (31 %) died on the emergency ward. Four of the ED stabilised patients were immediately transferred to another hospital. Of the remaining 164 patients, 63 patients died in hospital, on average 2 days after OHCA. After stabilisation on the CCU/ICU 36 patients were transferred to another hospital. In total 105 patients (43 %) survived to hospital discharge. Their hospital stay was on average 9 days. Three were discharged to a nursing facility and 66 patients went home.

### Factors associated with survival

Table 1 shows the characteristics of the 105 patients who survived until hospital discharge and the 137 patients who did not. Survivors were significantly younger, significantly more often had a cardiac arrest in a public area, a witnessed arrest, an arrest of cardiac origin and a shockable rhythm, AED was more often used, a shorter time until ROSC, a GCS ≥13 during ambulance transport post CPR and a longer length of hospital stay. No significant associations with survival were found for gender, bystander CPR, number of shocks and witnessed monitored cardiac arrest.

In the multivariate analyses, the variables location and use of AED were left out because of lack of independent association in the bivariate models. The time until ROSC was removed because of its almost linear relationship with the dependent variable survival. Logistic regression shows that four of the variables contribute significantly to surviving OHCA: witnessed cardiac arrest, cardiac cause, initial rhythm and GCS ≥13 (Table 2).

**Table 2:** Logistic regression on the likelihood of survival in OHCA patients

	p-value	Odds ratio	95 % CI for odds ratio	
			Lower	Upper
Age	0.068	0.975	0.950	1.002
Witnessed CA	0.019	2.851	1.187	6.849
Cardiac cause of arrest	0.009	5.947	1.569	22.549
ICR shockable	0.017	2.887	1.205	6.917
GCS	0.000	1.498	1.202	1.867

*CA* Cardiac arrest, *C.I.* Confidence interval, *GCS* Glasgow coma scale, *ICR* Initial cardiac rhythm

### Follow-up

For 78 of the 105 patients who survived until hospital discharge, follow-up data of at least 1 year after cardiac arrest were available. Of these patients 72 (92 %) were alive at least 1 year after cardiac arrest. Six patients died whereas the medical records of 27 patients were not available. The main reason patients were lost to follow-up was that they were living outside the Leiden region. Of the 72 survivors the average survival time was 28.6 months (SD 6.3) after the cardiac arrest 23 patients survived to 0–24 months, 38 patients survived to 24 and 36 months and 11 patients were still alive >36 months after cardiac arrest. All 6 patients who died after hospital discharge did so within a month after their cardiac arrest (5–25 days after cardiac arrest).

## Discussion

This study shows that a survival rate until hospital discharge of 43 % of EMS-treated OHCA patients is feasible in an optimised chain of survival. The survival rate is higher than the average 14 % as reported in Europe [1, 2]. However, a study in the Netherlands by Waalewijn et al.[3] also reported a high survival rate (36 %) of EMS-attended OHCA, be it that that study only included OHCA of cardiac origin whereas this study included all EMS-treated patients. In approximately 70 % of the cases the EMS decided to transport patients to the hospital (unpublished data), which is comparable with the 60 % EMS treatment found in the literature [1].

In this study survivors were younger, more often had a witnessed cardiac arrest, a cardiac origin of the arrest and a shockable rhythm compared with those who died. As expected, non-comatose patients (GCS ≥13 post CPR) and patients with sustained ROSC in the ambulance or ED had better chances of survival [4].

To find an explanation for the high survival rate, a comparison with the patient characteristics and treatment in the literature was made. The mean age (64.8 year) of the patients in the present study was in the same range as those seen in previous studies (64–67 years) [3, 5, 6]. In our study a relatively high proportion (67 %) of the OHCAs were witnessed and this was positively correlated with survival. We postulate that witnessing an OHCA contributes more to survival than CPR itself, since witnessed arrests give a higher chance of early alarm and use of an AED within minutes after the collapse.

A cardiac cause of cardiac arrest with an initial shockable rhythm may also partly explain the favourable outcome of this study. A systematic review reported that patients with VF or VT had a survival chance of 1 in every 4 to 7 patients compared with only 1 in every 21 to 500 patients in whom the first rhythm was asystole [4]. In the current study, a high percentage (49 %) of patients showed an initial cardiac rhythm of VF, whereas other studies reported that only 30 % of the patients had an initial rhythm of VF [9]. Since VF is only recorded in the acute stage after cardiac arrest, a high percentage of VF might indicate short arrival times of the ambulance service.

The use of an on-site AED doubles survival, probably caused by the reduction in time to first shock [9]. Berdowski found that an AED was used in 21 % of the cases [10]. In the current study AED was used in 39 % of the cases. It is plausible that the availability of AEDs has grown in the last 5 to 10 years, contributing to higher survival rates.

Another factor that might positively influence survival is the regional function of the LUMC for all cardiac arrests. Studies have described that lower mortality is seen in hospitals that treat a high volume of cardiac arrests and who provide 24 h interventional cardiac services [5, 6].

In this study 86 % of the patients who reached the CCU or ICU received on average 1.4 (sub)acute interventions per person. A comparison with the literature was not possible, since no studies on incidence of interventions in a comparable group were found. It is plausible that only patients with fair chances of survival receive interventions.

Of the 73 patients who underwent PCI 55 survived until hospital discharge and 18 died in hospital. Of the 12 coronary artery bypass graft procedures one patient died in hospital. Of the 26 patients who received an ICD during initial hospitalisation and the 34 patients who received an ICD after hospital discharge two patients died within a year after the cardiac arrest. The positive influence of an ICD in reducing mortality in the long term is described in a clinical review by Arrawwawala et al. [8]. Of the 95 patients who did not receive any intervention 80 died before hospital discharge. Follow-up data of 74 % of the patients were available, suggesting that after an average follow-up of 28.6 months the large majority were alive. However, as the medical records from many patients were not available, results have to be interpreted with caution.

All EMS-treated survivors of OHCA with a cardiac cause need cardiac rehabilitation. A recent Dutch study concluded that only 29 % of the patients eligible for cardiac rehabilitation received cardiac rehabilitation [11]. With increasing survival rates probably more effort should be put into aligning the process of cardiac rehabilitation for OHCA survivors.

## Conclusions

This study showed a survival rate of 43 % after OHCA in a urban region in the Netherlands where an optimised chain of acute and sub-acute treatment exists.

Witnessed cardiac arrest, cardiac origin of the arrest, shockable initial rhythm and GCS >13 post CPR were independently related to survival to hospital discharge. Availability of AED, short arrival times of EMS and (sub)acute treatment may also contribute to the success rate, but more research into the extent of the effect on survival is needed.
